# Productivity at work and quality of life in patients with rheumatoid arthritis

**DOI:** 10.1186/s12891-015-0562-x

**Published:** 2015-05-06

**Authors:** Myrthe van Vilsteren, Cecile RL Boot, Dirk L Knol, Dirkjan van Schaardenburg, Alexandre E Voskuyl, Romy Steenbeek, Johannes R Anema

**Affiliations:** Department of Public and Occupational Health, EMGO Institute for Health and Care Research, VU University Medical Center, Amsterdam, the Netherlands; Body@Work, Research Center Physical Activity, TNO-VU University Medical Center, Amsterdam, the Netherlands; Department of Epidemiology and Biostatistics, EMGO Institute for Health and Care Research, VU University Medical Center, Amsterdam, the Netherlands; Jan van Breemen Research Institute Reade, Amsterdam, the Netherlands; Department of Rheumatology, VU University Medical Center, Amsterdam, the Netherlands; TNO Work, Health and Care, Leiden, The Netherlands; Research Center for Insurance Medicine AMC-UMCG-UWV-VU University Medical Center, Amsterdam, the Netherlands

**Keywords:** Rheumatoid arthritis, At-work productivity loss, Quality of life

## Abstract

**Background:**

The aim of this study was to determine which combination of personal, disease-related and environmental factors is best associated with at-work productivity loss in patients with rheumatoid arthritis (RA), and to determine whether at-work productivity loss is associated with the quality of life for these patients.

**Methods:**

This study is based on cross-sectional data. Patients completed a questionnaire with personal, disease-related and environmental factors (related to the work environment), and clinical characteristics were obtained from patient medical records. At-work productivity loss was measured with the Work Limitations Questionnaire, and quality of life with the RAND 36. Using linear regression analyses, a multivariate model was built containing the combination of factors best associated with at-work productivity loss. This model was cross-validated internally. We furthermore determined whether at-work productivity loss was associated with quality of life using linear regression analyses.

**Results:**

We found that at-work productivity loss was associated with workers who had poorer mental health, more physical role limitations, were ever treated with a biological therapeutic medication, were not satisfied with their work, and had more work instability (R^2^ = 0.50 and R^2^ following cross-validation was 0.32). We found that at-work productivity loss was negatively associated with health-related quality of life, especially with dimensions of mental health, physical role limitations, and pain.

**Conclusions:**

We found that at-work productivity loss was associated with personal, work-related, and clinical factors. Although our study results should be interpreted with caution, they provide insight into patients with RA who are at risk for at-work productivity loss.

## Background

Rheumatoid arthritis (RA) is a chronic inflammatory disease with an impact on daily activities such as work [1]. The impact on work can be profound since permanent work disability (inability to continue working), is common among patients with RA [2]. In addition to the consequences for the patient, such as a decreased quality of life, work disability also leads to high costs. Approximately one-third of the total cost for patients with RA is caused by production losses [3]. Production losses include both lost work hours and times when patients are working, but their ability to meet work demands is limited (at-work productivity loss). Recently, the greatest impact on costs for patients with RA was shown to be reduced performance while working (at-work productivity loss), followed by wage loss from quitting or changing jobs, decreased working hours, and missed work days (sick leave) [4]. This implies that at-work productivity loss is an important concern, since work hours are not only lost incidentally through sick leave, but also more structurally and profoundly by at-work productivity loss.

Predictors for permanent work disability have been studied extensively and highlight the importance of personal factors such as education level and age, as well as disease-related factors such as perceived health complaints and limitations in daily activities caused by the disease [5,6]. In contrast, predictors for sick leave and at-work productivity loss have not been well researched, although interest in at-work productivity loss has been increasing since 49% of patients with RA have this experience [4]. The importance of at-work productivity loss has been recognized by the OMERACT (Outcome Measures in Rheumatology) initiative [7] and recently, measures for at-work productivity loss have been identified and validated [8-10]. Since measures for at-work productivity loss are now available, it is possible to investigate the work functioning of patients with RA. Until now, little has been known about the at-work productivity loss of this patient population [11]. Knowledge about factors associated with at-work productivity loss is needed to prevent both this loss and the possibility of leaving employment permanently in the future for patients with RA [12]. Therefore, it is vital to identify patients with RA who are at risk for at-work productivity loss in an early stage.

Prolonging work participation reflects an important contribution to fulfilling societal roles. It has been suggested that becoming permanently work-disabled is associated with a poorer quality of life in general [13-15]. Since patients who experience at-work productivity loss are prone to develop sick leave and permanent work disability in the future, we hypothesize that at-work productivity loss is associated with a low quality of life. This study has two research questions:

1. Which combination of personal, disease-related and environmental factors is best associated with at-work productivity loss in patients with RA?
2. Is at-work productivity loss associated with a low quality of life in patients with RA?

## Methods

### Design

This is a cross-sectional study of the baseline measurements of a randomised controlled trial (RCT) evaluating an intervention program to support at-work productivity for workers with RA. The RCT design is described in detail elsewhere [16]. Patients with RA were recruited from a specialized rheumatology clinic (Reade, formerly the Jan van Breemen Institute), regional hospitals (outposts of Reade), and an academic hospital (the VU University Medical Center, department of rheumatology), Amsterdam, the Netherlands.

Inclusion criteria for this study were: (1) a diagnosis of RA, (2) ages 18-64 years, (3) having a paid job (either paid- employment or self-employed), (4) working at least eight hours per week, (5) and experiencing at least minor difficulties in functioning at work. We asked patients to indicate on a scale of one to five the extent that RA interfered with their work functioning. Patients who indicated one on this scale, had a severe comorbidity, were unable to read or understand Dutch, or had had more than three months of sick leave at the time of inclusion were excluded. The study was approved by the medical ethics committees of the participating centres (Slotervaart Hospital, Reade, and the VU University Medical Center), and all patients gave written informed consent.

### Measurements

Patients completed a questionnaire and their clinical characteristics were obtained from their medical records. All measured variables were categorized into either internal (personal or disease-related variables) or external factors related to the external environment.

#### Outcome measures

At-work productivity loss was measured by hours lost from work due to presenteeism. Presenteeism is defined as being present at the job, but not being able to function optimally. Presenteeism was measured using the 25-item Work Limitations Questionnaire (WLQ) [17]. The WLQ includes four dimensions; physical demands, time management, mental-interpersonal demands, and output demands [18]. Based on these four dimensions, a score was calculated to represent the percentage of productivity loss. This score was multiplied by the number of work hours in two weeks, leading to an estimation of the number of hours that a patient had not been fully productive at work over the last two weeks. The internal consistency is high for the WLQ, with a Cronbach’s alpha of 0.88 [19]. Several studies showed that the WLQ is a reliable and valid questionnaire for assessing productivity loss in workers with RA [18-20].

Quality of life was measured using the RAND 36 [21,22]. All nine subscales of the RAND 36 were included in the questionnaire, and used for further analyses including mental health (Cronbach’s α 0.85), pain (α 0.88), physical role limitations (α 0.90), physical functioning (α 0.92), social functioning (α 0.71), vitality (α 0.82), emotional role limitations (α 0.86), general health perception (α 0.81), and perceived health change (α not applicable). The subscales of the RAND 36 are transformed into a scale score ranging from 0-100. A higher score indicates better health. The subscales of the RAND 36 are included as internal factors in the analysis.

#### Internal factors

The Disease Activity Score of 28 joints (DAS28) was assessed as part of usual care and was collected from patient records. The DAS28 score was based on the number of tender and swollen joints out of 28 joints, the erythrocyte sedimentation rate (ESR) and the patient’s general health measured on a visual analogue scale (VAS) of 100 mm [23]. We retrieved the use of disease-modifying antirheumatic drugs (DMARD) and the use of biological therapeutics. Serological measures (anti-cyclic citrullinated peptide antibodies [aCCP] and IgM rheumatoid factor [IgM-RF]) were recorded as positive or negative.

Daily functioning was measured with the Health Assessment Questionnaire (HAQ), a reliable and valid questionnaire widely used in RA research [24]. The HAQ score ranges from 0-3; a higher score indicates increased disability.

Pain and fatigue were measured with single items using VAS scales [25,26]. VAS scales range from 0-10, with 0 meaning no pain/ fatigue at all, and 10 meaning severe pain/ very tired.

The presence of comorbidity (yes/no) was investigated using a list with 15 common comorbidities including cardiovascular diseases, diabetes mellitus, and psychological disorders. Disease duration was determined by one open-ended question about the year of the patient’s RA diagnosis, and the duration of complaints due to RA (answer categories were 0-2 years, 3-5 years, 6-10 years, >10 years). We included one question about the highest attained educational level. Low education was operationalized as primary school, middle education or basic vocational education. Middle education was operationalized as secondary vocational education or intermediate vocational education. High education was operationalized as higher vocational education or a university degree. The patients’ gender and age were collected from patient medical records.

#### External factors

We measured supervisor and co-worker support, psychological and physical job demands with subscales from the Job Content Questionnaire (JCQ) [27]. Items of the JCQ such as ‘My colleagues are friendly’ are answered on a scale of 1 (totally disagree) to 4 (completely agree). The score of each subscale ranges from 1-4 with a higher score indicating more supervisor support, co-worker support, etc.

Work instability was measured with the RA Work Instability Scale (RA WIS) [28,29]. The RA WIS contains 23 items (yes/no), which are summed for a total score ranging between 0-23. A score of 0-9 indicates low, 10-17 moderate, and 18-23 high work instability.

Sick leave was measured with a single item of the Productivity and Disease Questionnaire (Prodisq) [30]. Patients were asked to count the number of days they had been absent from work during the last 12 months.

Three single item questions about being a supervisor (yes/no), shift work (yes/no), and type of job contract (permanent contract/self-employed) were included in the questionnaire worded as The Netherlands Working Conditions Survey [31].

### Statistical analyses

For the first aim, we determined which combination of factors was best associated with the outcome of at-work productivity loss using linear regression models. These models were built on a dataset of 100 randomly selected participants. The remaining 50 patients were used to cross-validate our model.

In step 1, we performed univariate linear regression analyses for each independent variable with at-work productivity loss. We did not include the number of working hours as an independent variable, since the number of work hours is already incorporated in the WLQ score for at-work productivity loss. Independent variables with a p-value <0.15 were selected for further analyses [32]. In step 2, we built a multivariate model for the nine subscales of the RAND 36, using a backward stepwise procedure, because the subscales are highly correlated. Similarly we built a backward multivariate model for the DAS 28 score and its four components that were highly correlated as well. We retained the components with a p-value <0.15 for further analyses.

In step 3, we built two multivariate models; one multivariate model for the selected internal variables and one model for the selected external variables. All independent variables selected in step 1 and 2 were entered in the models, using a backward stepwise procedure. Variables with a p-value <0.15 were retained in the multivariate models, resulting in an internal and external model. In step 4, one final model was built in which all variables retained in the internal and external model in step 3 were entered again using a backward procedure. Variables with a p-value <0.05 were retained in the final model. R^2^ was used to calculate the proportion of explained variance.

The final model was cross-validated in the remaining sample of n = 50 by calculating the R^2^ of the final model applied to the sample of n = 50. R^2^ was calculated by determining the correlation between the actual outcome measured with the questionnaire, and the outcome calculated based on the regression coefficients in the final multivariate model of step 4 (i.e. outcome calculated = constant + B1 * var1, etc.). The R^2^ of both samples (n = 100 and n = 50) were compared.

For the second aim of this study, univariate linear regression analyses were performed to gain insight into the crude associations between at-work productivity loss and quality of life. The independent variables were each subscale of the RAND 36 retained in the backward procedure from step 2, and the dependent variable was at-work productivity loss. Second, we investigated potential confounders for each univariate model. All variables with a p-value <0.15 with at-work productivity loss were considered as potential confounders. Confounders were selected based on a stepwise forward strategy. Each potential confounder was entered separately into the model. The strongest confounder based on the change in the regression coefficient was retained in the model, and subsequently, all remaining potential confounders were entered one by one. The strongest confounder was retained from each round, until the regression coefficient did not change significantly by adding the next confounder. A change in the regression coefficient of more than 10% was considered significant [32].

All analyses were performed in SPSS version 20.

## Results

Baseline measurements were available for 150 participants. Tables 1, 2 and 3 describe the characteristics of the study population. In general, women participated in this study (84.0%). The mean age of the study population was 49.7 years, and on average, our participants had been diagnosed with RA 10.4 years ago. The overall WLQ score was 7.1, meaning that 7.1% of the time during the past two working weeks had been lost due to lost work productivity. This finding led to an average estimate of 4.0 work hours lost due to presenteeism over two weeks, based on an average work week of 28.7 hours. The study population scored low on the RAND 36 scales for vitality, physical role limitations, general health perception, and perceived health change scales, indicating that they had worse health on these subscales compared to the other RAND 36 subscales.

**Table 1:** **Association of internal factors with at-work productivity loss for workers with RA**

**Baseline characteristics N = 150**		**At-work productivity loss**		
		**Univariate N = 150**		
	**Mean (sd)**	**B**	**95% CI**	**p-value**
*Internal factors*				
Age	49.7 (8.6)	-0.03	-0.08; 0.03	0.36
Female	84.0^a^	-0.83	-2.12; 0.45	0.20
Education:			Overall p-value:	0.29
Low	21.3^a^	Ref.	Ref.	Ref.
Middle	32.0^a^	-0.02	-1.28; 1.23	0.87
High	46.7^a^	0.71	-0.47; 1.89	0.24
Time since diagnosis	10.4 (8.9)	-0.03	-0.08; 0.02	0.25
Duration of complaints >10 years	47.3^a^	0.04	-0.87; 0.95	0.93
Comorbidity present	64.7^a^	-0.17	-1.12; 0.78	0.73
Pain (0-10^b^)	3.72 (2.50)	0.18	-0.002; 0.36	0.05
Fatigue (0-10^b^)	4.59 (2.53)	0.27	0.10; 0.44	<0.01
Health Assessment Questionnaire (0-3^b^)	0.79 (0.55)	0.69	-0.14; 1.53	0.10
Quality of life dimensions (RAND 36) (0-100^b^):				
- Mental health	77.2 (14.5)	-0.10	-0.13; -0.08	<0.01
- Pain	64.6 (17.8)	-0.05	-0.07; -0.02	<0.01
- Physical role limitations	48.0 (40.3)	-0.03	-0.04; -0.02	<0.01
- Physical functioning	67.1 (21.5)	-0.02^c^	-0.04; 0.002	0.08
- Social functioning	71.4 (21.4)	-0.06^c^	-0.08; -0.04	<0.01
- Vitality	54.3 (18.5)	-0.06^c^	-0.08; -0.04	<0.01
- Emotional role limitations	78.6 (37.1)	-0.03^c^	-0.04; -0.02	<0.01
- General health perception	50.8 (17.3)	-0.04^c^	-0.06; -0.01	<0.01
- Perceived health change	51.7 (26.7)	-0.02^c^	-0.04; -0.01	0.01

^a.^Expressed as n(%).

^b.^Range of the scale on which the score was based.

^c.^Item was not retained based on the second analyses step (multivariate models for the RAND36 subscales).

**Table 2:** **Association of clinical factors with at-work productivity loss for workers with RA**

**Baseline characteristics N = 150**		**At-work productivity loss**		
		**Univariate N = 150**		
	**Mean (sd)**	**B**	**95% CI**	**p-value**
*Clinical factors*				
DAS score 28 joints	2.70 (1.23)	0.57	0.20; 0.94	<0.01
- Swollen joints	1.01 (2.01)	0.19^c^	-0.04; 0.41	0.10
- Tender joints	2.11 (3.10)	0.14^c^	-0.01; 0.28	0.06
- ESR	12.8 (13.0)	0.03^c^	-0.004; 0.06	0.09
- VAS general health (0-100^b^)	34.5 (22.5)	0.03^c^	0.01; 0.05	0.01
Number of DMARDS used since diagnosis	2.13 (1.05)	0.29	-0.15; 0.72	0.19
Number of DMARDS used during last year	1.10 (0.70)	0.19	-0.49; 0.87	0.58
Biological therapeutic used since diagnosis; yes	47.3^a^	0.86	-0.05; 1.77	0.06
Biological therapeutic used during last year; yes	34.5^a^	0.34	-0.62; 1.31	0.48
RF positive	61.7^a^	0.08	-0.86; 1.02	0.87
aCCP positive	65.1^a^	0.13	-0.81; 1.07	0.79
DAS: Disease Activity Score; ESR: Erythrocyte Sedimentation Rate; VAS: Visual Analogue Scale; DMARD: Disease-modifying Antirheumatic Drug; RF: Rheumatoid Factor; aCCP: Anti-cyclic Citrullinated Peptide Antibodies.				

^a.^Expressed as n(%).

^b.^Range of the scale on which the score was based.

^c.^Item was not retained based on the second analyses step (multivariate model for the DAS components).

**Table 3:** **Association of external factors with at-work productivity loss for workers with RA**

**Baseline characteristics N = 150**		**At-work productivity loss**		
		**Univariate N = 150**		
	**Mean (sd)**	**B**	**95% CI**	**p-value**
*External factors*				
Permanent or temporary job contract	82.7^a^	-1.24	-2.41; -0.08	0.04
Being a supervisor (yes)	27.5^a^	0.98	-0.03; 2.00	0.06
Multiple jobs (yes)	16.8^a^	-0.04	-1.16; 1.08	0.95
Shift work (yes)	18.8^a^	1.07	-0.08; 2.21	0.07
Limited in functioning at work:			Overall p-value:	<0.01
No	13.3^a^	Ref.	Ref.	Ref.
Slightly	76.0^a^	2.14	0.85; 3.42	<0.01
Strongly	10.7^a^	2.66	0.89; 4.44	<0.01
Supervisor social support (1-4^b^)	2.99 (0.62)	0.35	-0.34; 1.03	0.32
Decision authority (1-4^b^)	2.70 (0.36)	0.24	-1.03; 1.49	0.71
Psychological job demands (1-4^b^)	2.65 (0.31)	-0.04	-1.54; 1.47	0.96
Physical job demands (1-4^b^)	1.98 (0.61)	0.63	-0.11; 1.37	0.09
Co-worker social support (1-4^b^)	3.09 (0.46)	0.22	-0.77; 1.21	0.67
Not or moderately satisfied with work	31.3^a^	2.35	1.47; 3.24	<0.01
Work instability (0-23^b^)	8.87 (4.80)	0.28	0.20; 0.36	<0.01
Sick leave during last 12 months		0.46	0.17; 0.75	<0.01
Heavy demanding work	70.7^a^	1.01	0.04; 1.98	0.04
Work demands:			Overall p-value:	0.25
Mental	66.7^a^	Ref.	Ref.	Ref.
Both physical and mental	21.3^a^	0.99	-0.18; 2.15	0.10
Physical	12.0^a^	0.11	-1.29; 1.52	0.87
Treatment center:			Overall p-value:	0.12
Specialized rheumatology center	76.7^a^	Ref.	Ref.	Ref.
Academic hospital	10.0^a^	-0.94	-2.40; 0.52	0.21
Regional hospitals	13.3^a^	-1.22	-2.54; 0.10	0.07

^a.^Expressed as n(%).

^b.^Range of the scale on which the score was based.

Following univariate regression analyses (Tables 1, 2 and 3), we built multivariate models for internal and external factors. In the internal model, we retained mental health, pain, physical role limitations and biological therapeutic use since diagnosis for further analyses. In the external model, we retained the variables for having a job contract, being a supervisor, limitations in functioning at work, job satisfaction, work instability, heavy and demanding work, and having both physical and mental job demands for further analyses.

The final model (Table 4) shows that participants who experienced worse mental health and more physical role limitations, who had been treated with a biological therapeutic since diagnosis, who were not or only moderately satisfied with their work, and had a higher work instability score experienced more at-work productivity loss. The R^2^ for this model was 0.50. The R^2^ of this model in the cross-validation sample was 0.32.

**Table 4:** **Factors associated with at-work productivity loss for workers with RA**

**Factor**	**Multivariate N = 100**	
	**B**	**95% CI**
Mental health	-0.06	-0.09 : -0.02
Physical role limitations	-0.01	-0.03; -0.002
Biological therapeutic used since diagnosis; yes	1.06	0.25; 1.87
Not or moderately satisfied with work	1.00	0.08; 1.92
Work instability	0.10	0.001; 0.21

For the second aim of our study, more at-work productivity loss was significantly associated (ß -0.10, 95% CI -0.13; -0.08) with worse mental health (Table 5). The adjusted analysis showed a regression coefficient of -0.06 (95% CI -0.09; -0.03), meaning that more at-work productivity loss was associated with worse mental health. The crude analysis of at-work productivity and the quality of life dimension for physical role limitations had a regression coefficient of -0.03; the adjusted analysis had a regression coefficient of -0.01. This means that on the physical role limitations scale (0-100, where a higher score indicates fewer role limitations) for every 10 points lower on the scale, an additional 0.1 hours are lost due to presenteeism. At-work productivity loss was significantly associated (ß -0.05, 95% CI -0.07; -0.02) with more pain. The adjusted analysis showed a regression coefficient of -0.03, 95% CI -0.06; 0.003), meaning that more at-work productivity loss is associated with more pain.

**Table 5:** **Association between at-work productivity loss and quality of life for workers with RA**

**Independent variables (Quality of life dimensions RAND 36):**	**Dependent variable: at-work productivity loss**			
	**Crude analysis**		**Adjusted analysis**	
	**ß**	**95% CI**	**ß**	**95% CI**
Mental health	-0.10	-0.13; -0.08	-0.06^a^	-0.09; -0.03
Physical role limitations	-0.03	-0.04; -0.02	-0.01^b^	-0.02; 0.0004
Pain	-0.05	-0.07; -0.02	-0.03^c^	-0.06; 0.003

^a.^Adjusted for work instability and job satisfaction.

^b.^Adjusted for work instability, mental health, HAQ, pain (RAND 36), and job contract.

^c.^Adjusted for work instability, pain (VAS), physical role limitations, mental health, and HAQ.

## Discussion

In this study, we showed that workers with RA lose 4.0 hours of productive work per two weeks on average due to at-work productivity loss based on an average work week of 28.7 hours. At-work productivity loss for workers with RA is associated with both internal and external factors. Workers with worse mental health and more physical role limitations reported more lost hours at work due to presenteeism, as did workers who had ever been treated with a biological therapeutic, who were not satisfied with their work, and those with more work instability. We also showed that at-work productivity loss is negatively associated with health-related quality of life, especially in the dimensions of mental health, physical role limitations, and pain. This means that a lower quality of life on these subdomains is associated with more lost hours due to presenteeism.

### Comparison with other studies

Only a few studies have reported on factors associated with at-work productivity loss for workers with RA. In a study by Geuskens et al. on workers with early inflammatory joint conditions, it was shown that at-work productivity loss was predicted by low support from colleagues, intermediate levels of pain, poor physical functioning and poor mental health [11]. Gignac et al. studied job disruptions (operationalized as limitations to meet work demands) in workers with inflammatory arthritis or osteoarthritis [12]. Job disruptions were associated with male sex, previous absenteeism, job change, and arthritis-work spill over (the extent to which the demands of arthritis interfered with work and work interfered with managing arthritis). In line with these studies, we found both internal and external factors related with at-work productivity. Our results show that at-work productivity loss was not only associated with physical functioning, but also with mental health, as was also found by Geuskens et al [11].

Only one clinical factor was retained in our final model in this study (use of biological therapeutics). We found that patients who had used a biological therapeutic since their disease was diagnosed experienced more at-work productivity loss. Although a recent review showed that the use of biological therapeutics had a potentially beneficial effect on work participation [33]. Longitudinal studies included in this review generally concerned patients with high disease activity or DMARD failure. In our study, we did not select patients based on indicators of disease activity. Our findings suggest that the use of biological therapeutics may not only be considered as more effective medication for patients with higher disease activity [34-36], but also as a marker of more severe disease. According to this criterion, patients in the present study probably have a higher than average disease severity, since the use of biologicals ever was 47.3%, whereas the total population with RA at Reade has a present biological use of 25% (according to internal communication with rheumatologists of the participating centres).

Two external factors were associated with at-work productivity loss: work instability and low job satisfaction. Work instability indicates a mismatch between functional ability and job demands [28]. In addition to the association of more work instability with more at-work productivity loss in our study, work instability was also shown to predict work transitions for patients with RA in another study [37]. Work transitions include reductions in work hours, sickness absence, job changes, and temporary employment. These findings, as well as our study results, show the potential prognostic value of the variable work instability on work-related outcomes. The importance of job satisfaction for work participation has been documented. In a study on chronic low back pain, moderate or poor job satisfaction predicted longer work absences [38].

We found that at-work productivity loss was associated with low quality of life. This association was most profound on the mental health dimension, indicating that worse mental health is associated with more at-work productivity loss, which is in line with previous research [11]. An earlier study on patients with psoriatic arthritis showed that work-disabled patients experience worse mental health compared to working patients [14]. This finding indicates that actually having a paid job has a positive influence on mental health. In our study of working patients with RA, we found that the mean scale score on the mental health scale of the RAND 36 was comparable to the Dutch normal value for this subscale [22]. Although our population on average did not have impaired on mental health, mental health remains a topic of concern, since for our patients with RA, worse mental health was associated with more at-work productivity loss.

We validated our multivariate model in our population (internal validation) [39]. In previous studies, multivariate models have been mainly used for dichotomous outcomes (i.e. sick/ not sick). For dichotomous multivariate models, two studies performed an internal validation of their model [40,41]. They compared, amongst others, R^2^ values that were comparable (<10% change in R^2^) between the multivariate model and validation model [40,41]. For prediction models with continuous outcomes, there are no validation studies available to our knowledge. Our R^2^ in the main sample was 0.50, and 0.32 in our validation sample. This decline indicates that the factors best associated with at-work productivity loss should be interpreted cautiously.

### Strengths and limitations

A strength of our study was that we included a variety of both internal and external factors in our analyses, instead of focusing on clinical factors only, for example. The average number of work hours lost due to presenteeism is in line with previous research. In a study by Zhang et al. who also measured at-work productivity loss with the WLQ, 4.0 hours were lost every two weeks due to presenteeism, although the average number of work hours was 35 hours per week in their study, compared to 28.7 hours in our study [42]. This supports the representativeness of our study sample.

A limitation is that our results are based on cross sectional data, therefore, we cannot distinguish causes and consequences.

Our results concern workers with RA who experienced at least minor difficulties at work. We were therefore not able to study the incidence of presenteeism.

We assessed sick leave with a long recall period, which can introduce recall bias. From previous research however, it seems that self-reports of sick leave are accurate when compared to sick leave registries [43].

### Study implications

Further research should focus on work functioning, and predictors for at-work productivity loss in cohort (longitudinal) studies to identify those patients most at risk.

Our results give an indication of risk factors for at-work productivity loss, but further investigation is needed for more robust findings. Since patients experiencing at-work productivity loss are at risk for sick leave and permanent work disability, it is important to know which factors contribute to at-work productivity loss, in order to develop interventions to improve at-work productivity loss and thereby prevent work disability. We included a broad range of potential factors, such as personal factors, clinical characteristics, and work-related factors in our analysis. Our multivariate model shows that the factors best associated with at-work productivity loss are drawn from all three domains. Future research should consider the potential impact of both personal and work-related factors in addition to clinical characteristics.

The importance of personal and work-related factors should also be acknowledged in current clinical practice if the goal is to enhance work participation. This means that treating physicians should not only focus on improving disease severity when treating patients with RA who are struggling to maintain work productivity, but they should also pay attention to personal- and work-related factors for a more holistic approach.

## Conclusions

In conclusion, we found that at-work productivity loss was associated with personal, work-related, and clinical factors for patients with RA. Low at-work productivity was associated with a lower quality of life, especially in the domains mental health, physical role limitations and pain. Although our study results should be interpreted with caution, they provide insight on which patients with RA are at risk for at-work productivity loss.

## Abbreviations

ACCP: Anti-cyclic citrullinated peptide antibodies
DAS28: Disease activity score of 28 joints
DMARD: Disease modifying antirheumatic drugs
ESR: Erythrocyte sedimentation rate
HAQ: Health assessment questionnaire
IgM-RF: IgM rheumatoid factor
JCQ: Job content questionnaire
Prodisq: Productivity and disease questionnaire
RA: Rheumatoid arthritis
RA WIS: Work instability scale
RCT: Randomized controlled trial
VAS: Visual analogue scale
WLQ: Work limitations questionnaire

## References

[1] Ahlstrand I, Bjork M, Thyberg I, Borsbo B, Falkmer T (2012). Pain and daily activities in rheumatoid arthritis. Disabil Rehabil.

[2] Boonen A, Severens JL (2011). The burden of illness of rheumatoid arthritis. Clin Rheumatol.

[3] Lundkvist J, Kastang F, Kobelt G (2008). The burden of rheumatoid arthritis and access to treatment: health burden and costs. Eur J Health Econ.

[4] Zhang W, Anis AH (2011). The economic burden of rheumatoid arthritis: beyond health care costs. Clin Rheumatol.

[5] De Croon EM, Sluiter JK, Nijssen TF, Dijkmans BA, Lankhorst GJ, Frings-Dresen MH (2004). Predictive factors of work disability in rheumatoid arthritis: a systematic literature review. Ann Rheum Dis.

[6] Detaille SI, Heerkens YF, Engels JA, van der Gulden JW, van Dijk FJ (2009). Common prognostic factors of work disability among employees with a chronic somatic disease: a systematic review of cohort studies. Scand J Work Environ Health.

[7] Tang K, Escorpizo R, Beaton DE, Bombardier C, Lacaille D, Zhang W (2011). Measuring the impact of arthritis on worker productivity: perspectives, methodologic issues, and contextual factors. J Rheumatol.

[8] Beaton DE, Tang K, Gignac MA, Lacaille D, Badley EM, Anis AH (2010). Reliability, validity, and responsiveness of five at-work productivity measures in patients with rheumatoid arthritis or osteoarthritis. Arthritis Care Res (Hoboken).

[9] Tang K, Beaton DE, Boonen A, Gignac MA, Bombardier C (2011). Measures of work disability and productivity: rheumatoid arthritis specific work productivity survey (WPS-RA), workplace activity limitations scale (WALS), work instability scale for rheumatoid arthritis (RA-WIS), work limitations questionnaire (WLQ), and work productivity and activity impairment questionnaire (WPAI). Arthritis Care Res (Hoboken).

[10] Tang K, Beaton DE, Lacaille D, Gignac MA, Bombardier C (2013). Sensibility of five at-work productivity measures was endorsed by patients with osteoarthritis or rheumatoid arthritis. J Clin Epidemiol.

[11] Geuskens GA, Hazes JM, Barendregt PJ, Burdorf A (2008). Predictors of sick leave and reduced productivity at work among persons with early inflammatory joint conditions. Scand J Work Environ Health.

[12] Gignac MA, Cao X, Lacaille D, Anis AH, Badley EM (2008). Arthritis-related work transitions: a prospective analysis of reported productivity losses, work changes, and leaving the labor force. Arthritis Rheum.

[13] Stigmar KG, Petersson IF, Joud A, Grahn BE (2013). Promoting work ability in a structured national rehabilitation program in patients with musculoskeletal disorders: outcomes and predictors in a prospective cohort study. BMC Musculoskelet Disord.

[14] Wallenius M, Skomsvoll JF, Koldingsnes W, Rodevand E, Mikkelsen K, Kaufmann C (2009). Work disability and health-related quality of life in males and females with psoriatic arthritis. Ann Rheum Dis.

[15] Chorus AM, Miedema HS, Boonen A, van Der Linden S (2003). Quality of life and work in patients with rheumatoid arthritis and ankylosing spondylitis of working age. Ann Rheum Dis.

[16] van Vilsteren M, Boot CR, Steenbeek R, van Schaardenburg D, Voskuyl AE, Anema JR (2012). An intervention program with the aim to improve and maintain work productivity for workers with rheumatoid arthritis: design of a randomized controlled trial and cost-effectiveness study. BMC Public Health.

[17] Lerner D, Amick BC, Rogers WH, Malspeis S, Bungay K, Cynn D (2001). The work limitations questionnaire. Med Care.

[18] Lerner D, Reed JI, Massarotti E, Wester LM, Burke TA (2002). The work limitations Questionnaire's validity and reliability among patients with osteoarthritis. J Clin Epidemiol.

[19] Walker N, Michaud K, Wolfe F (2005). Work limitations among working persons with rheumatoid arthritis: results, reliability, and validity of the work limitations questionnaire in 836 patients. J Rheumatol.

[20] Allaire SH (2003). Measures of adult work disability. Arthritis Rheum.

[21] Moorer P, Suurmeije T, Foets M, Molenaar IW (2001). Psychometric properties of the RAND-36 among three chronic diseases (multiple sclerosis, rheumatic diseases and COPD) in The Netherlands. Qual Life Res.

[22] Van der Zee KI, Sanderman R. Het meten van de algemene gezondheidstoestand met de RAND-36 : een handleiding. 1993. Groningen, Noordelijk Centrum voor Gezondheidsvraagstukken, Rijksuniversiteit Groningen.

[23] Burkhard FL, Andel I, Sautner J, Fassl C, Nothnagl T, Rintelen B (2007). The disease activity score in 28 joints in rheumatoid arthritis and psoriatic arthritis patients. Arthritis & Rheumatism.

[24] Bruce B, Fries JF (2003). The Stanford health assessment questionnaire: a review of its history, issues, progress, and documentation. J Rheumatol.

[25] Ho K, Spence J, Murphy MF (1996). Review of pain-measurement tools. Ann Emerg Med.

[26] Wolfe F (2004). Fatigue assessments in rheumatoid arthritis: comparative performance of visual analog scales and longer fatigue questionnaires in 7760 patients. J Rheumatol.

[27] Karasek R, Brisson C, Kawakami N, Houtman I, Bongers P, Amick B (1998). The Job content questionnaire (JCQ): an instrument for internationally comparative assessments of psychosocial job characteristics. J Occup Health Psychol.

[28] Gilworth G, Chamberlain MA, Harvey A, Woodhouse A, Smith J, Smyth MG (2003). Development of a work instability scale for rheumatoid arthritis. Arthritis Rheum.

[29] Gilworth G, Emery P, Gossec L, Vliet Vlieland TP, Breedveld FC, Hueber AJ (2009). Adaptation and cross-cultural validation of the rheumatoid arthritis work instability scale (RA-WIS). Ann Rheum Dis.

[30] Koopmanschap MA (2005). PRODISQ: a modular questionnaire on productivity and disease for economic evaluation studies. Expert Rev Pharmacoecon Outcomes Res.

[31] Van den Bossche SNJ, Koppes LLJ, Granzier JJM, De Vroome EEM, Smulders PGW. Netherlands working conditions survey 2007. Methods and general results. 2008. Hoofddorp, TNO Quality of Life.

[32] Twisk J (2007). Inleiding in de toegepaste biostatistiek.

[33] Ter Wee MM, Lems WF, Usan H, Gulpen A, Boonen A (2012). The effect of biological agents on work participation in rheumatoid arthritis patients: a systematic review. Ann Rheum Dis.

[34] Emery P, Breedveld FC, Hall S, Durez P, Chang DJ, Robertson D (2008). Comparison of methotrexate monotherapy with a combination of methotrexate and etanercept in active, early, moderate to severe rheumatoid arthritis (COMET): a randomised, double-blind, parallel treatment trial. Lancet.

[35] Smolen J, Landewe RB, Mease P, Brzezicki J, Mason D, Luijtens K (2009). Efficacy and safety of certolizumab pegol plus methotrexate in active rheumatoid arthritis: the RAPID 2 study. A randomised controlled trial. Ann Rheum Dis.

[36] Moreland LW (2004). Drugs that block tumour necrosis factor: experience in patients with rheumatoid arthritis. Pharmacoeconomics.

[37] Tang K, Beaton DE, Gignac MA, Lacaille D, Zhang W, Bombardier C (2010). The Work Instability Scale for rheumatoid arthritis predicts arthritis-related work transitions within 12 months. Arthritis Care Res (Hoboken).

[38] Heymans MW, Anema JR, van Buuren S, Knol DL, Van Mechelen W, de Vet HC (2009). Return to work in a cohort of low back pain patients: development and validation of a clinical prediction rule. J Occup Rehabil.

[39] Steyerberg EW, Vickers AJ, Cook NR, Gerds T, Gonen M, Obuchowski N (2010). Assessing the performance of prediction models: a framework for traditional and novel measures. Epidemiology.

[40] van de Stadt LA, Witte BI, Bos WH, van Schaardenburg D (2013). A prediction rule for the development of arthritis in seropositive arthralgia patients. Ann Rheum Dis.

[41] Bansback N, Young A, Brennan A, Dixey J (2006). A prognostic model for functional outcome in early rheumatoid arthritis. J Rheumatol.

[42] Zhang W, Gignac MA, Beaton D, Tang K, Anis AH (2010). Productivity loss due to presenteeism among patients with arthritis: estimates from 4 instruments. J Rheumatol.

[43] Linton SJ (2011). Correspondence of back pain patients' self-reports of sick leave and Swedish National Insurance Authority register. Percept Mot Skills.

